# *MEIS1*, *PREP1*, and *PBX4 *Are Differentially Expressed in Acute Lymphoblastic Leukemia: Association of *MEIS1 *Expression with Higher Proliferation and Chemotherapy Resistance

**DOI:** 10.1186/1756-9966-30-112

**Published:** 2011-12-20

**Authors:** Judith A Rosales-Aviña, Jorge Torres-Flores, Adriana Aguilar-Lemarroy, Carmen Gurrola-Díaz, Georgina Hernández-Flores, Pablo C Ortiz-Lazareno, José M Lerma-Díaz, Ruth de Celis, Óscar González-Ramella, Esperanza Barrera-Chaires, Alejandro Bravo-Cuellar, Luis F Jave-Suárez

**Affiliations:** 1 División de Inmunología, Centro de Investigación Biomédica de Occidente - IMSS, Sierra Mojada No. 800, CP 44340, Guadalajara, Jalisco, Mexico; 2 Instituto de Enfermedades Crónico Degenerativas, Departamento de Biología Molecular y Genómica, Centro Universitario de Ciencias de la Salud, Universidad de Guadalajara, Sierra Mojada No. 900, CP 44340, Guadalajara, Jalisco, Mexico; 3 Laboratorio de Inmunología, Departamento de Fisiología, Centro Universitario de Ciencias de la Salud, Universidad de Guadalajara, Sierra Mojada No. 950, CP 44340, Guadalajara, Jalisco, Mexico; 4 Servicio de Hematología, Hospital Civil de Guadalajara Fray Antonio Alcalde, Hospital No. 278, CP 44280, Guadalajara, Jalisco, Mexico

**Keywords:** TALE genes, leukemia, MEIS1, PREP1, PBX, Apoptosis

## Abstract

**Background:**

The Three-amino acid-loop-extension (*TALE*) superfamily of homeodomain-containing transcription factors have been implicated in normal hematopoiesis and in leukemogenesis and are important survival, differentiation, and apoptosis pathway modulators. In this work, we determined the expression levels of *TALE *genes in leukemic-derived cell lines, in blood samples of patients with Acute lymphoblastic leukemia (ALL), and in the blood samples of healthy donors.

**Results:**

Here we show increased expression of *MEIS1, MEIS2, *and *PREP1 *genes in leukemia-derived cell lines compared with blood normal cells. High levels of *MEIS1 *and *PREP1*, and low levels of *PBX4 *expression were also founded in samples of patients with ALL. Importantly, silencing of *MEIS1 *decreases the proliferation of leukemia-derived cells but increases their survival after etoposide treatment. Etoposide-induced apoptosis induces down-regulation of MEIS1 expression or *PREP1 *up-regulation in chemotherapy-resistant cells.

**Conclusions:**

Our results indicate that up-regulation of *MEIS1 *is important for sustaining proliferation of leukemic cells and that down-regulation of *MEIS1 *or up-regulation of *PREP1 *and *PBX *genes could be implicated in the modulation of the cellular response to chemotherapeutic-induced apoptosis.

## Background

Three-amino-acid loop extension *(TALE) *genes belong to the homeobox group and are distinguished by the presence of three extra amino acids in the loop binding the first to the second alpha helix of the homeodomain [1]. TALE proteins include subfamilies MEINOX and PBC. MEINOX is composed of the members MEIS1, MEIS2, the recently described MEIS3, PREP1, and PREP2 in humans [1,2]. The PBC subfamily contains PBX1, PBX2, PBX3, and PBX4 proteins [3,4]. Expression of *TALE *genes has been related with normal development, differentiation, survival, apoptosis, and with the hematopoietic process [5-10]. Indeed, some *TALE *genes are targets for viral insertion or for chromosome translocations during leukemogenesis. In this regard, *MEIS1 *has been characterized as a common proviral integration site in BXH-2 mice [11]; in these mice, leukemic tumors that contain a viral integration site at the *MEIS1 *locus frequently possess an additional co-integration site in some *HOX *genes [12], which suggests the required cooperative effect of *MEIS *and *HOX *during leukemogenesis. Over-expression of *MEIS1 *in CD34^+ ^hematopoietic cells has been related with suppression of differentiation, promotion of proliferation, and self-renewal. Interestingly, high levels of *MEIS1 *in myeloid progenitors have been shown to regulate the cellular response to some cytokines, favoring self-renewal or differentiation. Moreover, in the murine myeloid cell line 32Dcl3, it has been observed that *MEIS1 *can block granulocytic differentiation in response to G-CSF [13]. *MEIS1 *has been also found over-expressed in human leukemic cells [14].

Other TALE proteins that have been also related with normal hematopoiesis and leukemogenesis comprise members of the PBX group. PBX proteins were first identified as HOX cofactors involved in developmental gene regulation [15,16]. *PBX1 *plays a role in the development of blood cell populations because hematopoietic stem cells from *PBX1*-/- embryos have reduced colony-forming activity and are unable to establish multilineage hematopoiesis in competitive reconstitution experiments [8]. PBX-PREP1 complexes are required for the production of normal CD4 and CD8 T-lymphocytes. Furthermore, PBX-MEIS complexes have been implicated in megakaryocyte differentiation, and PBX-PREP complexes have been also connected with the regulation of Interleukin (IL)-10 production in macrophages during the phagocytosis of apoptotic cells [17]. PREP proteins are also important during development; for instance, deletion of *PREP1 *in mice and zebrafish induces embryonic lethality [18,19]. Mice hypomorphic for *PREP1 *exhibit defects in T-cell development, with a decreased number of single-positive thymocytes, increased apoptosis of double-positive thymocytes, and abnormalities in the expression of αβ and γδ T-cell receptors [19]. Additionally, reduction in *PREP1 *expression directly affects the expression of *MEIS *and *PBX *and consequently, normal embryonic hematopoiesis [20].

In summary, *TALE *genes codify for important transcription factors involved in hematopoiesis and leukemogenesis and are important survival, differentiation, and apoptosis pathway modulators in hematopoietic cells. In this study, we analyzed the expression of *TALE *genes in leukemia-derived cell lines, in samples of patients with Acute lymphoblastic leukemia (ALL), and in samples of clinically healthy donors. We observed consistent up-regulation of *MEIS1 *and *PREP1 *and down-regulation of *PBX4 *in leukemic cell lines and in the samples of patients with ALL. Interestingly, RNA-mediated down-modulation of *MEIS1 *lowers leukemic cell proliferation. Additionally, chemotherapeutic treatment of lymphoblastic cell lines induces an increment in *PREP1*, *PBX2, *and *PBX4 *messenger RNA (mRNA) levels that could be related with a more resistant phenotype.

## Results

### Higher Expression Levels of *MEIS1*, *MEIS2*, and *PREP1 *Genes in Leukemia-derived Cell Lines Compared with Normal Cells

**Figure 1:**
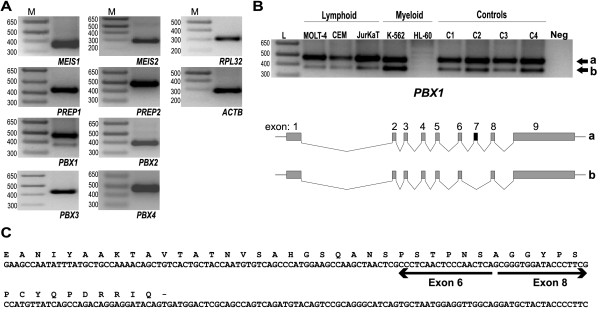
**Analysis of primers used for amplification of Three-amino-acid loop-extension *(TALE) *family member genes**. **A) **A pull of cDNA obtained from leukemia-derived cell lines was utilized to test the specificity and efficiency of each set of primers in the amplification of *TALE *family genes. After 40 cycles of amplification by conventional PCR, the PCR products were separated into 2% agarose gels and visualized under Ultraviolet (UV) light. Amplification products of the reference genes employed (*RPL32 *and *ACTB*) are also included. The 1 Kb Plus DNA Ladder (Invitrogen, Life Science) is shown in the left line; **B) **Amplification of *PBX1 *in leukemia-derived cell lines and in healthy controls separated into 2% agarose gels and visualized under UV light (upper panel). Genome map of complete (a) and alternative (b) splicing of *PBX1*; **C) **Sequence of alternative splicing of *PBX1 *showing adjacent coding regions of the deleted exon.

*TALE *genes are normally involved in the differentiation of hematopoietic cells and their expression has been related with deregulated differentiation. From this starting point, we wanted to determine the expression levels of *TALE *genes in cells with impaired differentiation. For this purpose, we choose leukemic-derived cell lines and normal differentiated cells as the study model. In order to analyze the expression of *TALE *genes, we first selected primer pairs to amplify these genes and proved these primers by conventional PCR reactions utilizing a pull of complementary DNA (cDNA) obtained from leukemia-derived cell lines (Table 1). All primer pairs were able to amplify a specific product corresponding to the expected size for each *TALE *gene (Figure 1A); however, for *PBX1, *we observed an additional band of lower molecular size. We carried out the same approach to analyze primer pairs designed to amplify the L32 ribosomal protein (*RPL32*) and Beta-Actin (*ACTB*) in order to have reference genes to measure relative gene expression (Figure 1A). Regarding PBX1, we also noticed the low-molecular-weight band existing in controls and in all leukemia derived cell lines (Figure 1B). By sequence analysis, we found that this corresponds to an isoform of *PBX1 *in which exon 7 is lost (Figures 1B and 1C). This indicated that our PCR primers were specific for *PBX1*, and also that they are able to amplify both *PBX1 *versions. Because we planned to quantify gene expression by real time PCR (qRT-PCR) utilizing the SYBR Green strategy, in the case of *PBX1*, the value obtained in quantification correspond to the sum of the expression of both isoforms.

**Table 1:** Primer pairs used for PCR reactions

GENE	ACCESION NUMBER	PRIMER SEQUENCE	Tm
***MEIS1***	[GenBank:NM_002398]	F: CCC CAG CAC AGG TGA CGA TGA T R: TGC CCA TTC CAC TCA TAG GTC C	60

***MEIS2***	[GenBank:NM_170677]	F: CCA TCG ACC TCG TCA TTG AT R: CCT CCT TTC TTC TGG CGT TTT T	60

***PREP1***	[GenBank:NM_004571]	F: GGT TTT GGC CTG ATT CTA TTG C R: GTG GGG AGG GAG TGG TG	65

***PREP2***	[GenBank:NM_022062]	F: GCC ACC AAT ATA ATG CGT TCT T R: GTG TTC CAA GCC CAG GTC	65

***PBX1***	[GenBank:NM_002585]	F: CTA ACT CGC CCT CAA CTC C R: GTG TCC AGA TTG GCT GAA ATA G	60

***PBX2***	[GenBank:NM_002586]	F: GGC GGC TCT TTC AAT CTC TCA R: GTC TCG TTA GGG AGG GGA TGA C	65

***PBX3***	[GenBank:NM_006195]	F: CAA GGG TCC CAA GTC GG R: TGG CCT AAT TGG ATA AAG TGC T	60

***PBX4***	[GenBank:NM_025245]	F: ATG GGG AAG TTT CAA GAA GAG G R: ATC TCG AGT CGC AGC AGA C	65

***GAPDH***	[GenBank:NM_002046]	F: CAC TGC CAC CCA GAA GAC TGT G R: TGT AGG CCA TGA GGT CCA CCA C	60

***RPL32***	[GenBank:NM_000994]	F: GCA TTG ACA ACA GGG TTC GTA G R: ATT TAA ACA GAA AAC GTG CAC A	60

***ACTB***	[GenBank:NM_001101]	F: TCC GCA AAG ACC TGT ACG R: AAG AAA GGG TGT AAC GCA ACT A	60

**Figure 2:**
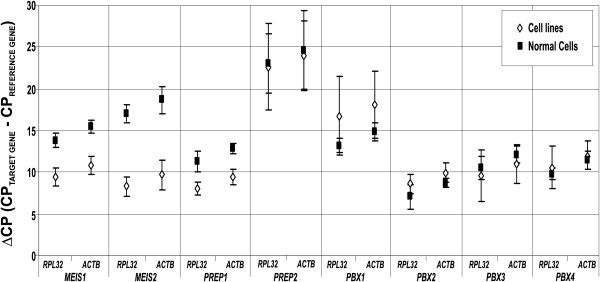
**Baseline expression level of Three-amino-acid loop-extension *(TALE) *family genes (*MEIS1*, *MEIS2*, *PREP1*, *PREP2*, *PBX1*, *PBX2*, *PBX3*, and *PBX4*) in healthy cells vs. leukemia-derived cell lines**. The graphics-display means and Standard deviation (SD) of ΔCP values obtained for the expression level of *TALE *genes. Values were calculated taking *RPL32 *or *ACTB *as reference genes. The squares and diamonds represent means ± SD of two independent experiments.

Next, we proceeded to analyze the expression of *TALE *genes by qRT-PCR in leukemia-derived cell lines. We employed five cell lines including Jurkat, CEM, MOLT-4, K562, and HL-60; the first three are lymphoblastic, and the latter two, myeloid. We determined the crossing point for each target gene and subsequently normalized this with the crossing point of an internal reference gene to calculate the ΔCP, which represents an absolute and more comparative value (see Materials and Methods). It is important to bear in mind that the ΔCP value is inversely proportional to gene expression. To obtain more consistent results, we use two different reference genes: *RPL32*, and *ACTB*. As can be observed in Figure 2, results obtained with *RPL32 *and *ACTB *follow the same tendency. In this regard, *RPL32 *and *ACTB *were selected as confident reference genes. Interestingly, when expression levels of *TALE *genes in leukemic cell lines and blood-derived normal cells were evaluated, significant differences were observed in the expression of *MEIS1, MEIS2, *and *PREP1*. Normal blood cells have greater ΔCP values for these three genes, thus lower expression (Figure 2). For *PREP2 *and all *PBX *members, we did not observe any variation. Additionally, on comparing ΔCP values we could note that in all cell lines and control cells, *PREP2 *possesses the lowest mRNA level.

### Up-regulation of *MEIS1 *and *PREP1 *and Down-regulation of *PBX4 *in ALL Samples vs. Those of Healthy Individuals

**Figure 3:**
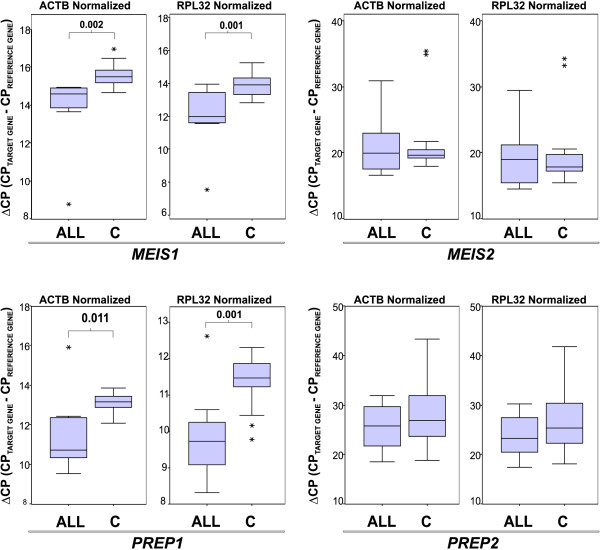
**Levels of *MEIS1*-*2 *and *PREP1*-*2 *in healthy volunteers vs. patients with leukemia**. Box plot graphics showing ΔCP values taking *ACTB *(left panel) or *RPL32 *(right panel) as reference genes. The graphics display median (dark lines), 25‒75^th ^percentile (boxes), interquartile ranges (whiskers), and outliers (*) from the 14 patients with Acute lymphoblastic leukemia (ALL) and the 19 controls (C). Unpaired Student *t *test was used to compare ALL with Control group. Statistical significances are shown between groups only when *p *≤ 0.05.

**Figure 4:**
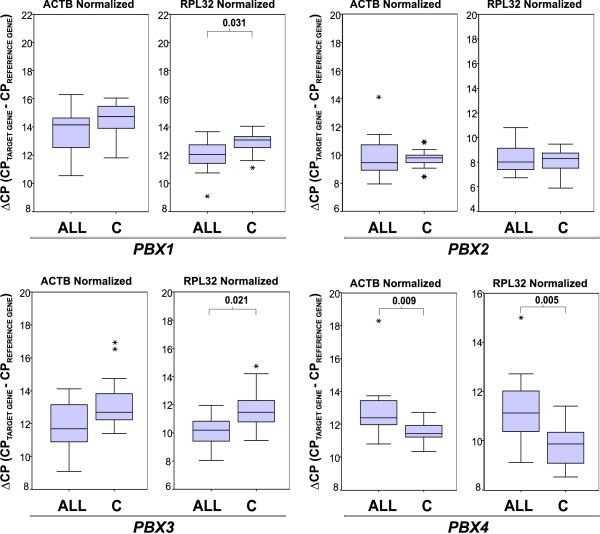
**Levels of *PBX1*-*4 *in healthy volunteers vs. patients with leukemia**. Box plot graphics showing ΔCP values taking *ACTB *(left panel) or *RPL32 *(right panel) as reference genes. The graphics display median (dark lines), 25‒75^th ^percentile (boxes), interquartile ranges (whiskers), and outliers (*) from the 14 patients with Acute lymphoblastic leukemia (ALL) and the 19 controls (C). Unpaired Student *t *test was used to compare ALL with Control group. Statistical significances are shown between groups only when *p *≤ 0.05

To confirm whether variations in TALE expression observed in cell lines were also observed in samples of patients with leukemia, we recruited 14 samples of patients diagnosed with Acute lymphoblastic leukemia (ALL) and 19 samples from clinically healthy volunteers (Table 2). We again analyzed the genetic expression of *TALE *genes by qRT-PCR employing the previously mentioned *RPL32 *and *ACTB *as reference genes to calculate ΔCP values. As can be observed in Figure 3, distribution of ΔCPs obtained for ALL samples were noticeably different from those obtained for control samples in the cases of *MEIS1 *and *PREP1*. Differences in ΔCP values for *MEIS2 *and *PREP2 *in patients compared with controls were not statistically significant. For the PBX group (see Figure 4), we observed that *PBX1 *and *PBX3 *were, to some extent, up-regulated in patients with ALL, but this difference was only statistically significant when we normalized with reference gene *RPL32*. *PBX2 *expression remained unchanged in patients and controls, and the sole member that clearly exhibited down-regulation in ALL samples was *PBX4*.

**Table 2:** Overview of controls and patients

Control ID	Gender	Age (years)	Patient ID	Gender	Age (years)	Diagnosis
**1**	M	33	1	M	38	ALL

**2**	M	26	2	M	82	ALL

**3**	F	54	3	M	56	ALL

**4**	F	34	4	F	46	ALL

**5**	F	68	5	F	32	ALL

**6**	M	51	6	F	36	ALL

**7**	F	43	7	F	56	ALL

**8**	F	24	8	M	84	ALL
**9**	F	56	9	M	61	ALL

**10**	M	40	10	M	58	ALL

**11**	F	53	11	F	30	ALL

**12**	F	35	12	M	52	ALL

**13**	F	26	13	F	43	ALL

**14**	M	39	14	M	18	ALL

**15**	M	73				

**16**	M	45				

**17**	F	39				

**18**	M	40				

**19**	M	26				

ALL, Acute lymphoblastic leukemia; ID, identification; M, Masculine; F, Feminine.

### *MEIS1 *Silencing Decreases the Proliferation Rate of Leukemic-derived Cell Lines

**Figure 5:**
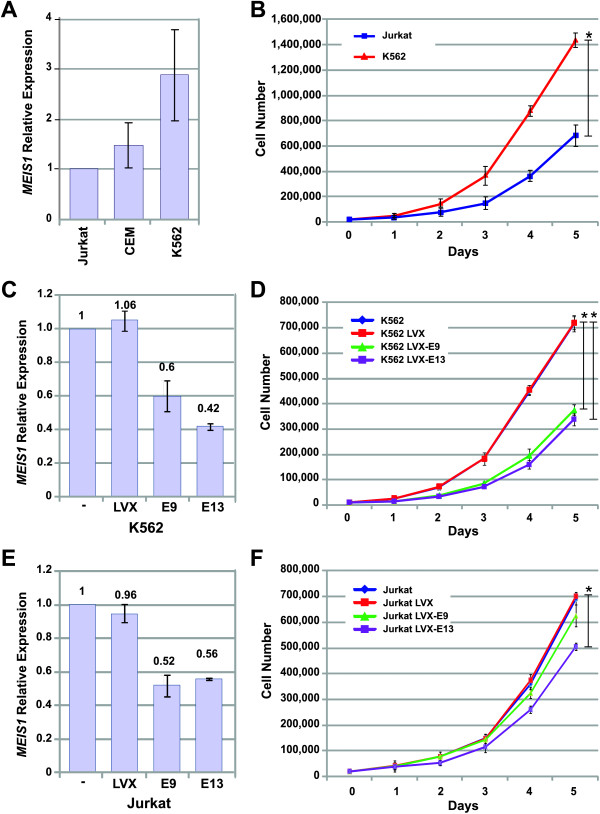
**Effect of *MEIS1 *expression on cell growth of leukemia-derived cell lines**. A) Expression levels of MEIS1 were analyzed by qRT-PCR in Jurkat, CEM, and K562 cells; expression of RPL32 was also determined and used as reference gene to calculate relative expression; B) Cell proliferation analysis of K562 and Jurkat cells; C, E) Expression levels of MEIS1 in Jurkat and K562 cell lines infected with virus carrying shRNA-E9 or shRNA-E13. Values were obtained by qRT-PCR using RPL32 as reference gene; D, F) Proliferation of *MEIS1*-silenced cells. Jurkat and K562 cells were infected with an shRNA directed to exon 9 (LVX-E9) and an shRNA directed to exon 13 (LVX-E13). Cell growth was determined counting the cells daily for 5 days. Graphics show means ± Standard deviations (SD) of values obtained from three independent experiments. Statistical differences were calculated at the end point of proliferation curves using 2 way ANOVA analysis and Bonferroni posttest, (*) significances are shown between groups only when *p *≤ 0.05.

Because we determined a consistent up-regulation of *MEIS1 *and *PREP1 *in cell lines and in samples of patients with ALL, it was interesting to us to determine which type of advantage provides the high expression of these genes to leukemic cells. First we analyzed the role of *MEIS1*. The *MEIS1 *gene has been localized in chromosome 2 and it has been described that Jurkat cells are monosomic for this chromosome, CEM cells have two copies, and K562 cells are trisomic [21]; in this regard, expression of *MEIS1 *ought to be different in these cell lines. To test this hypothesis, we analyzed *MEIS1 *baseline expression in these cell lines by qRT-PCR (Figure 5A). As expected, Jurkat was the cell line with the lowest *MEIS1 *expression, followed by CEM and K562 expressing highest levels. Taking advantage of the existing different levels of *MEIS1 *in the cell lines, we utilized Jurkat and K562 cells to investigate whether high *MEIS1 *expression is related with increased proliferation. We observed that K562 have a higher proliferation rate than Jurkat cells (Figure 5B). To demonstrate the direct involvement of *MEIS1 *in this exacerbated proliferation, we performed silencing assays in both cell lines. We employed short hairpin RNAs shRNAs directed to two different regions of *MEIS1 *mRNA: one was directed to Exon 9 (E9), and the other to Exon 13 (E13). By using recombinant virus, we introduced these sequences into Jurkat and K562 cells. To assure that all infected cells were carrying the construction, a resistance gene to puromycin was also introduced and the infected cells were selected with this antibiotic. Additionally, we infected the cells with an empty virus (without shRNA) and selected them also with puromycin in order to posses a control for the selection and infection process. We then tested *MEIS1 *(mRNA) levels by qRT-PCR. As shown in Figures 5C and 5E, *MEIS1 *mRNA levels decrease with both shRNAs in both cell lines to nearly 50% of the initial expression. Employing the *MEIS1*-silenced cells, we then measured the proliferation rate and observed that proliferation was affected in all clones in which *MEIS1 *was silenced (Figures 5D and 5F). This effect was more obvious in K562 cells, which are the cells that express more *MEIS1*.

### Expression of *MEIS1 *and *PREP1 *Is Modulated in Response to Apoptosis Induction by Etoposide

**Figure 6:**
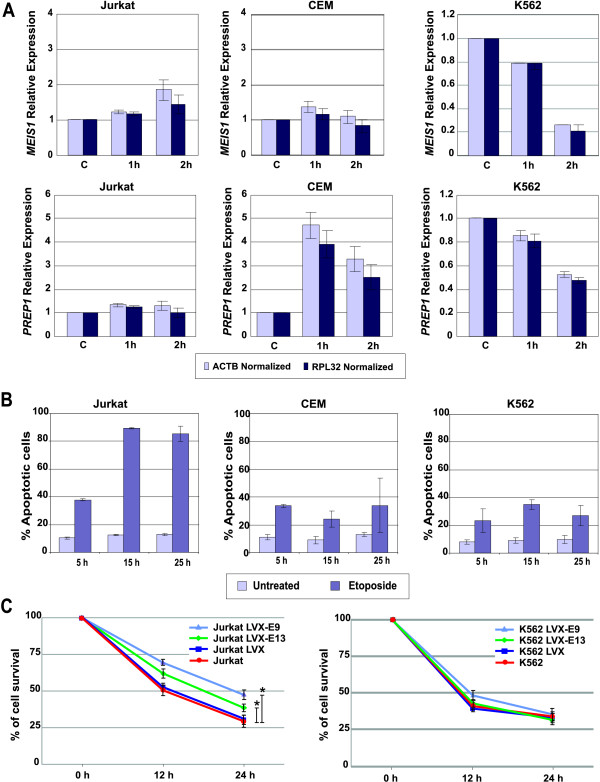
**Modulation of *MEIS1 *and *PREP1 *expression after etoposide treatment**. A) Jurkat, CEM, and K562 cells were treated with 170 μM etoposide for 1 and 2 h; thereafter, total RNA was extracted and retrotranscribed. Real time-PCR assays were performed to determine the relative expression levels of *MEIS1 *and *PREP1*. Expression analysis was carried out by normalizing with non-treated cells and employing *RPL32 *as reference gene. The bars represent means ± Standard deviations (SD) of two independent experiments; B) Percentage of apoptotic cells induced by etoposide treatment. Jurkat, CEM, and K562 cells were treated with 170 μM etoposide; the percentage of apoptotic cells was measured using Annexin-V-FLUOS. The bars represent means ± Standard deviations (SD) of three independent experiments. C) MEIS1-silenced (LVX-E9 and -E13) cells were treated with 170 μM etoposide for 12 and 24 hours. Parental cells (Jurkat and K562) or empty vector-silenced cells (LVX) were also used. After etoposide treatment WST-1 was added to cell cultures and incubate for 3 additional hours. The percentage of cell survival was calculated measuring Optical density (OD) at 450 nm (OD of untreated cells was set as 100%). Statistical differences were calculated at the end point of the curves using 2 way ANOVA analysis and Bonferroni posttest, (*) significances are shown between groups only when *p *≤ 0.05.

The other *TALE *member that we found up-regulated in leukemic cells was *PREP1*. Expression of this gene has been associated with resistance to apoptosis and it also has been described that *PREP1 *regulates *MEIS1 *expression [20,22]. In this respect, we subsequently analyzed whether the expression of *PREP1 *and *MEIS1 *was related with resistance to apoptosis induction by chemotherapeutic stimulus in leukemic cells. In order to assess this parameter, cultured cells were exposed to etoposide for 1 or 2 h; thereafter, variations in *MEIS1 *and *PREP1 *expression were analyzed by qRT-PCR. We observed that after etoposide treatment, Jurkat cells exhibit a tendency to increase *MEIS1 *expression, CEM cells remained unchanged, while diminishes K562 expression was noteworthy (Figure 6A). For *PREP1*, nearly no difference was observed in Jurkat cells; the response of CEM cells was more important because a notorious up-regulation was evidenced. Interestingly, K562 cells down-regulate *PREP1 *expression in response to etoposide (Figure 6A). To correlate these observations with phenotypic response, we measured the percentage of apoptotic cells after 5, 15, and 24 h of etoposide treatment. As can be observed in Figure 6B, Jurkat cells were the cells most sensitive to etoposide action; in contrast, CEM and K562 cells were the most resistant cells.

Given that K562 cells show a chemotherapeutic-resistant phenotype and that response of these cells to etoposide exposure is the down-modulation of *MEIS1*, and because we observed that Jurkat cells increased *MEIS1 *expression and were the most sensitive cells, we postulate that *MEIS1 *down-regulation could be a mechanism for resistance to etoposide-induced apoptosis. In this regard, Jurkat clones with *MEIS1*-silenced should be more resistant than Jurkat infected with the empty virus (pLVX) or with parental Jurkat cells. We tested this hypothesis exposing the cells to etoposide and measuring the percentage of surviving cells (Figure 6C). From this approach, we observed that Jurkat clones in which *MEIS1 *was silenced demonstrated a higher percentage of cell survival compared with pLVX infected cells or parental cells. *MEIS1 *silencing in K562 cells did not further increased the percentage of surviving cells.

## Discussion

*TALE *genes are a particular group of homeobox genes that are important in the regulation of proliferation, apoptosis, and normal cell differentiation. Anomalous expression of these genes has been involved in the development of hematological malignancies [23]. In this work, we first analyzed variations in the expression of *TALE *genes in leukemia-derived cell lines compared with normal control cells. In that we observed dissimilar *MEIS1*, *MEIS2*, and *PREP1 *expression levels, we wished to confirm whether these changes were also observed in samples of patients with leukemia. Interestingly, we found variations in *MEIS1*, *PREP1*, and *PBX4 *expression. It has been reported that over-expression of *MEIS1 *blocks myeloid cell differentiation; thus, high levels of *MEIS1 *are required to maintain hematopoietic cells in an undifferentiated state [13]. We observed high levels of *MEIS1 *mRNA in leukemia-derived cell lines and also in the blood samples of patients with ALL; our results are in agreement with observations that lower levels of *MEIS1 *are unfavorable for cell life, because *MEIS1 *down-regulation has been related with decreased proliferation and poor survival of neuroblastoma and leukemia cell lines [24,25]. Moreover, Kawagoe *et al*. reported that down-regulation of *MEIS1 *is required to induce differentiation of hematopoietic cells [26]. Our findings support the notion that this gene plays an oncogenic role and that its expression is required to sustain proliferation and block differentiation in leukemia cells [24,27]. Controversially, it has been reported that high levels of this protein can also trigger apoptosis; we observed that high *MEIS1*-expressing K562 cells were more resistant to apoptosis induction than Jurkat cells, which exhibited lower levels of *MEIS1*; however, it is also well known that MEIS1 requires the presence of protein partners to achieve its different functions [16,28,29]; one explanation for the contradictory effects reported for MEIS1 could be that, regardless of higher MEIS1 expression, cells can regulate the action of this protein by modulating the expression of MEIS1 cofactors, such as HOX. The availability of the later can transform MEIS1 action from proliferative into pro-apoptotic [28].

In the cell lines studied, we observed that an apoptotic stimulus induces *MEIS1 *up- and down-regulation (Jurkat and K562, respectively). A strategy of tumor cells for survival could be down-regulation of MEIS1. In this respect, through lowering its proliferation rate, tumor cells avoid DNA damage, which can induce apoptosis.

Regarding *MEIS2 *expression, this gene has been found in immature neuronal precursor cells, lens proliferative cells, ovarian cancer, and other tumor cell types, which underlies its possible role in sustaining proliferation [30]. We observed strong expression in leukemia-derived cell lines compared with control cells, which is in agreement with the findings of Smith *et al. *[31]; however, when we analyzed its expression in patients, we found no variation in the expression of this gene (Figure 3).

To a greater extent, we observed that all studied cell lines express *PREP1*, but not *PREP2*. *PREP1 *has been described to be ubiquitously expressed in adult tissues [32] and *PREP2 *is depicted as possessing more restricted expression, being negative in peripheral blood leukocytes [2]. After apoptosis induction by etoposide, CEM cells greatly increase *PREP1 *gene expression, *PREP1 *has been directly involved in the regulation of apoptosis: it has been described that *BCL*_*XL*_, an intrinsic apoptotic-pathway regulator, is a direct target of *PREP1 *[22]. PREP proteins interact with PBX members to achieve their functions [33]. Interaction of PREP with PBX1 and PBX2 increases the stability of PBX proteins and additionally increases the affinity of PREP for DNA binding [34,35]; the expression of BCL_XL _and p53 has been reported to be regulated by PREP1 in cooperation with PBX1b [22,36]. In etoposide-treated CEM cells, it was observed that expression of *PBX2 *and *PBX4 *increases (Additional file 1); *PBX2 *has been reported as a negative apoptosis modulator through negative regulation of BCL2 [37]. Up-regulation of *PREP1 *together with down-regulation of *PBX *genes could account for the etoposide-resistant phenotype of CEM cells; this resistance could be mediated by BCL_XL _and p53. High levels of p53 have been associated with apoptosis but, in the presence of BCL_XL_-mediated survival signals, p53 can induce senescence instead of apoptosis [38].

## Conclusions

In conclusion, our study shows that MEIS1 and PREP1 mRNA levels are significantly up-regulated in patients with ALL in comparison with healthy controls and inversely, that *PBX4 *is down-regulated in patients with ALL. Importantly, utilizing silencing assays, we confirmed that down-modulation of *MEIS1 *produces a lower leukemic-cell proliferation rate, an effect that was most notorious in the K562 myeloblastic cell line. Etoposide- induced apoptosis leads to changes in the expression of *PREP1 *and *MEIS1*; up-regulation of *PREP1 *and down-regulation of *MEIS1 *were independently related with resistance to apoptosis. Taken together, these results support the important role that *TALE *genes play in leukemic cell proliferation and survival, in addition to their probable involvement during leukemia development. Therefore, it could be important to evaluate *MEIS1 *and *PREP1 *expression in patients with leukemia prior to and after chemotherapeutic treatment and to correlate these findings with the clinical response.

## Methods

### Cells and cell culture

We used five commercially available human leukemia-derived cell lines: MOLT-4; Jurkat and CEM cells derived from lymphoid leukemia; HL-60 derived from promyelocytic leukemia, and K562 from erythroleukemia. Cells were grown in RPMI-1640 medium supplemented with 10% Fetal bovine serum (FBS), penicillin (100 U/mL,) and streptomycin (100 μg/mL); all products mentioned previously were obtained from GIBCO™ (Invitrogen Corp., Carlsbad, CA, USA). Cultures were maintained at 37°C in a humidified atmosphere with 5% CO_2_.

### Patients and sample collection

Peripheral blood samples were collected from 14 patients with Acute lymphoblastic leukemia (ALL) according to World Health Organization (WHO) classification criteria at the Centro Médico Nacional de Occidente of the Mexican Social Security Institute (CIBO-IMSS) and the Hospital Civil de Guadalajara Fray Antonio Alcalde. Additionally, blood samples from 19 healthy donors were also collected from the IMSS Blood Bank. Letters of informed consent and protocols were approved by the CLIS-1305 Ethical Board of CIBO-IMSS.

### Drugs and *in vitro *cell treatments

Etoposide was obtained from Lemery Laboratorios, México. The drug was stored at 4°C for <4 days and adjusted to the desirable concentration with DMEM culture medium immediately prior to utilization. The concentration employed was 170 μM etoposide.

### RNA extraction and cDNA synthesis

Total RNA was isolated by using the PureLink™ Micro-to-Midi Total RNA Purification System (Invitrogen Corp.) from 5 × 10^6 ^cultured cells as described by the manufacturer. Peripheral blood samples from patients with leukemia and from healthy donors were collected with EDTA as anticoagulant and mixed immediately after collection with 45 mL of RNA/DNA Stabilization Reagent for Blood/Bone Marrow (Roche Applied Science, Germany) and stored at ‒80°C for preservation. The stabilized samples were utilized for mRNA isolation via a two-step procedure by means of magnetic separation employing the mRNA Isolation kit for blood/bone marrow (Roche Applied Science). mRNA was finally eluted from the magnetic pearls in 20 μL of water and stored at ‒80°C until use.

cDNA synthesis was performed from 5 μg of total RNA or 12 uL mRNA employing the Transcriptor First Strand cDNA Synthesis kit primed with oligo(dT) (cat. no. 04897030001, Roche Applied Science). The protocol was conducted as recommended by the manufacturer. cDNA were stored at ‒20°C and aliquots were utilized as templates for PCR and RT-PCR reactions.

### PCR and RT-PCR

PCR reactions were carried out utilizing the set of primers presented in Table 1; the primers were designed using Oligo v6.0 software from sequences obtained from the NCBI-website GenBank Nucleotide database. PCR was performed using Taq DNA Polymerase (cat. no. 11146173001, Roche Applied Science) and Deoxynucleoside triphosphates (cat. no. 1969064, Roche Applied Science) in a PX2 Thermal Cycler (Thermo Electron Corp.). All reactions were conducted in 20 μL at the specified Tm (see Table 1). PCR products were resolved in 2% agarose gels containing 0.1 μg/mL ethidium bromide (Sigma Aldrich, Germany), visualized under Ultraviolet (UV) light, and documented with a DigiDoc-It System, (UVP, UK). RT-PCR analysis was achieved by employing the LightCycler-FastStart DNA MasterPLUS SYBR Green I kit (cat. no. 03515885001, Roche Applied Science) in the LightCycler 1.5 System (Roche Diagnostics GmbH, Mannheim, Germany). Data were normalized to the expression of the reference genes *RPL32 *(L32 Ribosomal Protein) and *ACTB *(β-actin).

### ΔCP analysis

To normalize target gene expression, we employed two different reference genes. We calculated the Crossing point (CP) for target and reference genes in each sample and subsequently calculated the ΔCP value of each sample, i.e., the target gene CP minus the reference gene CP. This facilitated analysis by taking only the intrinsic values of each sample. CPs from *ACTB*, and *RLP32 *were employed for this analysis. It is extremely noteworthy that ΔCP is inversely proportional to the expression of the target gene.

### Lentivirus production and infection

Oligonucleotides used to construct shRNAs were: forward 5'- GAT CCG CGG GAC TCA CCA TCC TTC AAG TGA ATT CAA GAG ATT CAC TTG AAG GAT GGT GAG TCC CGT TTT TTG-3' and reverse 5'-AAT TCA AAA AAC GGG ACT CAC CAT CCT TCA AGT GAA TCT CTT GAA TTC ACT TGA AGG ATG GTG AGT CCC GCG-3' directed to *MEIS1 *exon 9 (E9); forward 5'-GAT CCG CCG TGT GTT TAG AAG CCT AAT TCA AGA GAT TAG GCT TCT AAA CAC ACG GCT TTT TTA CGC GTG-3 and reverse 5'- AAT TCA CGC GTA AAA AAG CCG TGT GTT TAG AAG CCT AAT CTC TTG AAT TAG GCT TCT AAA CAC ACG GCG-3' directed to *MEIS1 *exon 13 (E13); this latter sequence has been previously used [24]. Complementary primers were annealed and cloned into the vector pLVX-shRNA1 (Clontech Laboratories, USA) using the restriction sites BamHI and EcoRI (NEB-Biolabs, USA). To produce infectious viral particles, Lenti-X 293T cells were transient-transfected by Lentiphos HT/Lenti-X HT Packaging Systems with lentiviral vectors pLVX-Puro or pLVX-shRNA1-E9 or pLVX-shRNA1-E13 as described by the manufacturer (Clontech Laboratories, USA). After 48 h, supernatants were checked with Lenti-X GoStix (Clontech Laboratories, USA) to determine whether sufficient viral particles were produced before transducing target cells. Supernatants were filtered through a 0.22-μm PES filter to eliminate detached cells, were aliquoted, and subsequently stored at ‒80°C until use. Jurkat and K562 cells (2.5 × 10^5^) were transduced with approximately 4.5 × 10^5 ^IFU/mL of supernatants. RNA extractions were obtained after at least 2 weeks of puromycin selection (1 μg/mL).

### Cell survival determination

Cell survival was determined by cleavage of tetrazolium salt WST-1 to formazan by cellular mitochondrial dehydrogenase enzymes. After different treatment periods, cells were incubated with 10 μL/well of WST-1/ECS solution (BioVision Research, Mountain View, CA, USA) for 3 h. Absorbance (450 nm) of treated and untreated samples was determined on a microtiter plate reader (Synergy™ HT Multi-Mode Microplate Reader; Biotek, Winooski, VT, USA). Data are reported as percentage of cell survival taking untreated control cells as 100% of cell survival.

### Apoptosis detection

Cell death was measured by flow cytometry using propidium iodide (cat. no. P4864, Sigma-Aldrich) and Annexin-V-FlUOS (cat. no. 1828681, Roche Applied Science) as recommended by these manufacturers. Cells were seeded in 6-well plates at a density of 3 × 10^5 ^cells per well in 1 mL RPMI medium containing or not etoposide (170 μM). After 5, 15, and 25 h, each sample was analyzed in a FACS Aria cytometer (BD Biosciences).

## Competing interests

The authors declare that they have no competing interests.

## Authors' contributions

JAR-A, JT-F, and AA-L carried out the PCR experiments but were also involved in all of the experimental work. GH-F, PCO-L, and JML-D made up the cell culture and devised drug treatment and flow cytometry for apoptosis detection. RdC determined cell survival. AB-C, CG-D, OG-R, and EB-C were involved in the recruitment of patients with leukemia and controls. AA-L and LFJ-S performed the statistical analysis, conceived of and designed the study, and wrote the manuscript. All authors helped to draft the manuscript and in reading and approving this final version.

## Supplementary Material

Additional file 1 **Modulation of *PBX1-4 *expression after etoposide treatment**. Jurkat and CEM cells were treated with 170 μM etoposide for 1 and 2 h; thereafter, total RNA was extracted and retrotranscribed. Real time-PCR assays were performed to determine the relative expression levels of *PBX1-4*. Expression analysis was carried out by normalizing with non-treated cells and employing *RPL32 *as reference gene. The bars represent means ± Standard deviations (SD) of two independent experiments. Click here for file
