# Supplementary figures and images for: MEIS1, PREP1, and PBX4 Are Differentially Expressed in Acute Lymphoblastic Leukemia: Association of MEIS1 Expression with Higher Proliferation and Chemotherapy Resistance
Source: J Exp Clin Cancer Res. 2011 Dec 20;30(1):112. doi: 10.1186/1756-9966-30-112 (PMC3259065; doi:10.1186/1756-9966-30-112)

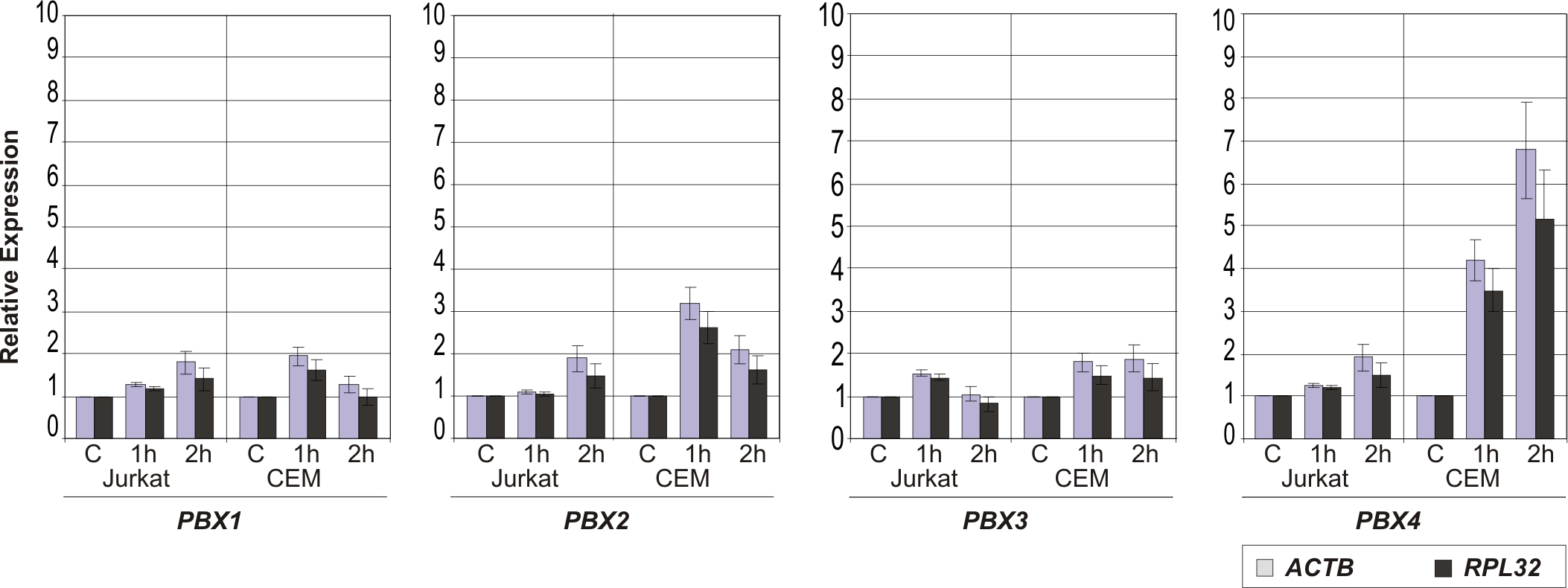

Supplement: Additional file 1 — Modulation of PBX1-4 expression after etoposide treatment. Jurkat and CEM cells were treated with 170 μM etoposide for 1 and 2 h; thereafter, total RNA was extracted and retrotranscribed. Real time-PCR assays were performed to determine the relative expression levels of PBX1-4. Expression analysis was carried out by normalizing with non-treated cells and employing RPL32 as reference gene. The bars represent means ± Standard deviations (SD) of two independent experiments. [file 1756-9966-30-112-S1.JPEG]
